# Assessing the reliability of baseline maximum voluntary contraction protocols

**DOI:** 10.7717/peerj.20848

**Published:** 2026-02-24

**Authors:** Gillian E. Slade, Michael W.B. Watterworth, Nicholas J. La Delfa

**Affiliations:** Health Science (Kinesiology), Ontario Tech University, Oshawa, Ontario, Canada

**Keywords:** Exercise science, Reliability, Strength, Baseline strength, Elbow, Knee, Hand grip

## Abstract

**Background:**

Maximum Voluntary Isometric Contractions (MVICs) are commonly used to normalize contraction intensity as a percentage of maximum; however, there is substantial variation in reported baseline MVIC protocols and no known consensus on their reliability. As such, the purpose of this study was to determine the method of baseline MVIC calculation that maximizes between-session reliability.

**Methods:**

Eighteen participants performed five knee extension, elbow flexion, and hand grip MVICs during four experimental sessions. Thirty-two methods of calculating baseline MVIC were evaluated using differing numbers of contractions, using the peak or average, and the presence of a familiarisation session and/or practice contraction. The level of significance was set at *p* ≤ 0.05 for all results presented. Reliability statistics were assessed across the 32 calculation methods, as was the effect of contraction and session number on MVIC strength.

**Results:**

Within-day Intraclass Correlation Coefficients (ICCs) estimates ranged from 0.94 to 0.98 for all contraction types and sessions, with between-day ICC estimates ranging from 0.85 to 0.99. Reliability marginally increased as more contractions were factored for both average and peak. Familiarisation and practice only improved reliability for elbow flexion. All baseline MVIC methods had acceptable between-day reliability. Multiple approaches to calculating baseline MVIC are reliable, but the most efficient method is to use the peak of one contraction. This approach balances high reliability with reduced participant fatigue and testing time, making it a practical option for both research and clinical applications.

## Introduction

Maximum isometric voluntary contractions (MVICs) represent the maximal force a muscle or muscle group can generate during an isometric contraction (Riemer, Jaitner & Wischniewski, 2023). The measurement of MVICs serves a vital role in biomechanics, ergonomics, and rehabilitation sciences. In most research contexts, MVICs provide a consistent modality for quantifying muscular strength and/or serving as a robust standard by which subsequent contractions can be normalised or compared to Boettcher, Ginn & Cathers (2008). By dividing a contraction force or torque by baseline MVIC strength, researchers can: (1) normalise contraction intensities across individuals during experiments, (2) better quantify strength changes between and within sessions, and (3) allow for inter-individual comparisons between those with wide-ranging strength capability.

Despite the availability of dynamic strength testing, isometric tests are often preferred in both clinical and ergonomics applications as they are highly correlated to dynamic strength (Nygard et al., 1994), use accessible equipment (Essendrop, Schibye & Hansen, 2001), and are easier to perform in a workplace setting. Clinically, measures of maximal strength can be used to assess a patient’s strength recovery status (Lagerström & Nordgren, 1996). One underlying assumption with MVICs is that individuals can produce maximal efforts with a high level of reliability between multiple measurements, as well as between days. However, even small day-to-day variability in baseline MVIC can impact results, especially when normalised strength values are used to define experimental workloads during a fatigue study (Abdel-Malek et al., 2022; Abdel-Malek et al., 2021), or used as threshold limit values in occupational design to limit excessive force demands (Essendrop, Schibye & Hansen, 2001; Potvin, 2012). Strength measurements may be substantially underestimated when MVIC tests lack familiarization or reliability controls. Although worst-case reductions of up to 20–30% have been reported in untrained individuals (Soderberg & Knutson, 2000), typical test–retest improvements in clinical and applied settings are closer to 5–25% (Eitzen, Hakestad & Risberg, 2012; Quittan et al., 2001; Selig et al., 2002). This underscores the importance of acquiring reliable and consistent MVICs.

Across the literature, researchers have reported numerous strategies to ensure consistency in MVIC measurements. However, there is currently no consensus on how many MVICs should be performed to calculate a reliable baseline maximal strength value. Researchers require participants to perform a varying number of contractions to calculate baseline MVIC strength throughout the literature, with studies employing one (Whittaker, Sonne & Potvin, 2019), two (La Delfa et al., 2022), three (Bao & Silverstein, 2005; Boettcher, Ginn & Cathers, 2008; Bohannon, 1986; Callaghan et al., 2000; Clarkson et al., 2005; Frost et al., 2012; Howatson & Van Someren, 2005; Hug et al., 2014; Kan, Dundas & Nosaka, 2013; Park & Hopkins, 2012; Strupstad, 2015), or five (Todd, Gorman & Gandevia, 2004) MVICs. A widely utilised method is to determine the average from three, 3-second MVICs (Brown & Weir, 2001). Previous studies have specifically used three MVICs due to how frequently it is found in published works (Frost et al., 2012). In contrast, selecting the peak strength among the performed MVIC(s) has also been widely reported (Abdel-Malek et al., 2021; Bao & Silverstein, 2005; Callaghan et al., 2000; Howatson & Van Someren, 2005; La Delfa et al., 2022; Strupstad, 2015; Todd, Gorman & Gandevia, 2004), and is argued to be more representative of maximal strength (Roberts et al., 2011). In addition to these differences, some researchers opt to include an entire familiarisation session (Abdel-Malek et al., 2022; Kan, Dundas & Nosaka, 2013; Strupstad, 2015), while others do not (Howatson & Van Someren, 2005; Peacock et al., 1981). Furthermore, some researchers use practice or warm-up contractions (Callaghan et al., 2000; Todd, Gorman & Gandevia, 2004), whereas other researchers do not report the use of any (Bao & Silverstein, 2005; Boettcher, Ginn & Cathers, 2008). While many presume a more reliable baseline can be captured with more exertions, conducting too many MVICs runs the risk of developing neuromuscular fatigue, defined as a decrease in force-generating capability (Enoka & Duchateau, 2016), which can clearly confound certain experimental designs.

The lack of a standardised procedure for obtaining and calculating baseline MVIC is perplexing, particularly given the availability of standardised procedures for other modalities, such as the number of contractions required for electromyography normalisation (Besomi et al., 2020). Given the absence of guidelines for determining baseline MVIC, the purpose of this study is twofold. The first aim is to examine the impact of familiarisation sessions, practice contractions, and the number of contractions on the between-day reliability of baseline MVIC measurements. By systematically investigating these factors, we seek to elucidate the key influences on between-day reliability of baseline MVIC assessments, and to offer insights for researchers and practitioners aiming to optimise the reliability of baseline MVICs across experimental and clinical settings. Secondly, we intend to assess the development of fatigue during MVIC assessments and evaluate the inherent within-day reliability of these measurements. Based on these objectives, we hypothesize that different methodologies will produce varying levels of between-session reliability, familiarisation sessions and warm-up contractions will increase day-to-day reliability, and maximal strength will progressively decrease as additional contractions are performed.

## Materials & Methods

A method comparison experimental design was used. Maximum strength values produced by participants were collected and were run through 32 different methods to calculate baseline strength. The primary objective was to conduct a reliability analysis on the various calculation methods to determine baseline MVIC strength. The secondary goal was to examine the effects of number of sessions, and number of contractions, on MVIC strength for elbow flexion, knee extension, and hand grip by conducting a repeated measures analysis. An a priori sample size check was conducted using previously established guidelines (Bujang & Baharum, 2017), which indicated that 4–5 participants are sufficient to estimate ICC values of 0.60–0.90 with acceptable precision; therefore, a sample of 18 participants was deemed adequate. In addition, a larger sample was required to implement a fully counterbalanced Latin square design, ensuring that all participants experienced the MVIC conditions in different orders and minimizing potential sequence or carryover effects.

### Subjects

Eighteen (nine males, nine females) participants were recruited from a convenience sample from a university student population. Exclusion criteria included a history of dominant upper or lower limb pain, injury, and/or surgery in the past 12 months. Additionally, participants were asked to refrain from resistance training prior to all data collection sessions, and to maintain a similar dietary/caffeine intake throughout their time participating. Participants completed the Edinburgh Handedness Inventory (Oldfield, 1971) and Waterloo Footedness Questionnaire (Melick et al., 2017) to determine handedness and footedness. This study was approved by the Ontario Tech University Research Ethics Board [#17071] and both written and verbal consent was obtained from all participants prior to study participation. Participant descriptors can be found in Table 1.

**Table 1:** Participant anthropometrics $(\bar {x}~\pm ~\sigma )$. Participant anthropometric characteristics (mean ± SD) separated by sex and combined total. Handedness and footedness are reported as counts of right- (R) and left- (L) dominant individuals.

	Age (years)	Height (cm)	Weight (kg)	Handedness	Footedness	Resistance training
Males (*n* = 9)	22.9 ± 2.47	178 ± 10.4	85.3 ± 17.0	8R 1L	9R 0L	5Y 4N
Females (*n* = 9)	19.8 ± 1.86	160 ± 15.9	71.4 ± 15.9	8R 1L	8R 1L	4Y 5N
Total (*n* = 18)	21.3 ± 2.66	169 ± 12.5	78.4 ± 17.5	16R 2L	17R 1L	9Y 9N

### Data acquisition

MVICs of the elbow and knee were collected using a HUMAC NORM isokinetic dynamometer (Computer Sports Medicine Inc., Stoughton, MA, USA) sampled at 2,500 Hz. The HUMAC NORM was adjusted to participant anthropometry for knee and elbow exertions. Measurements of the isokinetic dynamometer position were recorded for each participant to allow for replication at subsequent data collection sessions. During the first session, all setup parameters (*e.g.*, seat height and rotation, lever arm position, and dynamometer height) were adjusted to the individual and the corresponding scale values on the HUMAC NORM were documented. These recorded values were then used to replicate the identical positioning for that participant across all subsequent sessions. Hand grip MVICs were measured using a JAMAR Hydraulic Hand Dynamometer (Patterson Medical Inc., Warrenville, IL, USA) with peak force recorded in kilograms (kg). The JAMAR hand dynamometer was set at handle position two for all participants, as this position produced the most reliable results in previous literature (Fess, 1992; Gam et al., 2025; Massy-Westropp et al., 2011). A single experimenter conducted all JAMAR hand dynamometer and HUMAC NORM force measurements to eliminate potential biases arising from different raters.

### Experimental protocol

**Figure 1:**
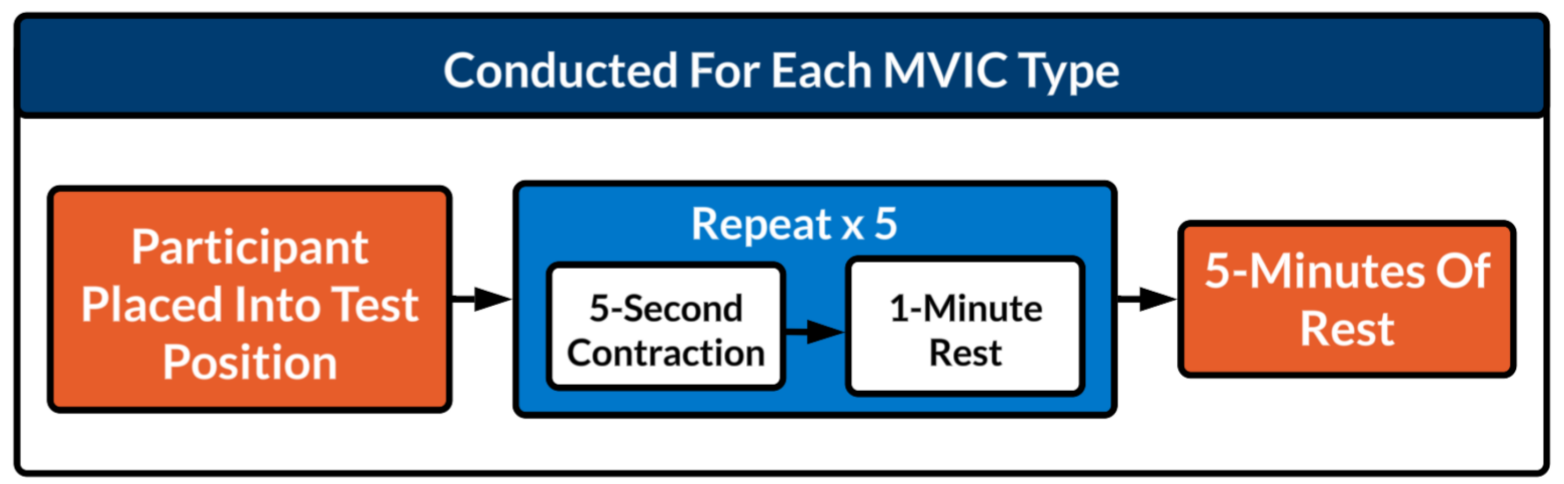
Within-day protocols. Schematic of the maximal voluntary isometric contraction (MVIC) testing protocol. For each MVIC type, participants were placed into the test position, performed five 5-second contractions separated by 1-minute rests, followed by a 5-minute rest before the next testing sequence.

Participants performed MVICs of the elbow, knee, and hand grip on four separate sessions, each at least 48 h apart. Each participant was scheduled at a similar time of day for all sessions to minimise diurnal variation in maximum strength performance (Knaier et al., 2022). Contraction type was randomised per session and per participant using a balanced Latin square to minimise task order effects. Within-day protocols are illustrated in Fig. 1.

**Figure 2:**
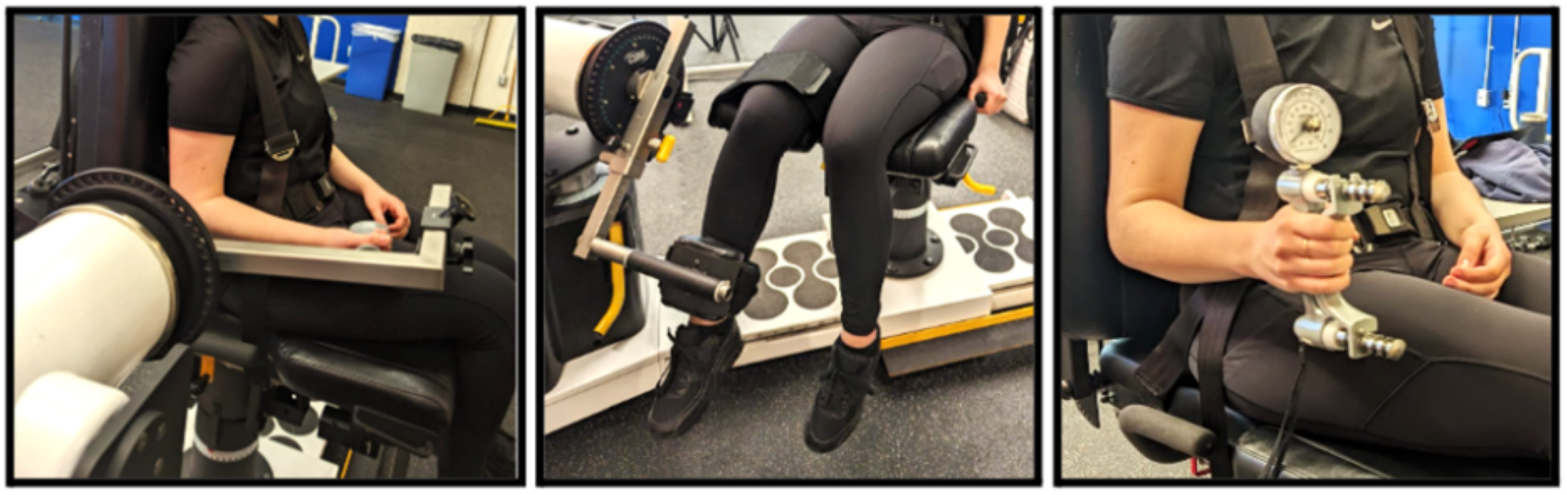
Participant posture for MVICs shown from left to right: elbow flexion, knee extension, and hand grip. Each panel illustrates a different isokinetic dynamometry setup used to measure muscle strength in various joints. Left: Shoulder abduction force assessment using a dynamometer arm positioned at shoulder level. Middle: Knee extension force measurement with the subject seated and the ankle secured to the machine arm. Right: Grip strength evaluation with the subject holding a handheld dynamometer. In all setups, subjects are stabilized using straps to ensure isolation of the target muscle group and minimize compensatory movements. Photo credit: Michael Watterworth.

Participants were secured into the HUMAC NORM using a 4-point seatbelt for all contraction postures (Fig. 2). The specific joint angles were selected based on previous literature and recommended testing positions. During elbow flexion MVICs, the elbow angle was set to 90° using a goniometer (Stobbe, 1982). For knee extension MVICs, the knee was positioned at 60° of flexion, a posture associated with high force-generating capacity along the knee torque–angle curve (Knapik et al., 1983). For both knee and elbow tasks, the joint centre was aligned with the dynamometer’s centre of rotation. Handgrip MVICs were performed using a JAMAR hand dynamometer. Participants remained secured in the HUMAC NORM seat and held the dynamometer at their side, with the elbow positioned at 90° and the forearm and wrist in a neutral posture, consistent with recommended handgrip testing procedures (Fess, 1992).

Participants were seated in the HUMAC NORM, which was properly adjusted to participants’ anthropometry (Fig. 2). Participants performed five MVICs with one minute of rest between each. A script was read before each set of contractions at each data collection session to keep instructions consistent between days. MVICs were instructed to be five seconds in duration, with a second of ramp-up and a second of ramp-down. The first contraction was treated as a practice contraction depending on the calculation method. In cases where a practice contraction was applied to the MVIC calculation, the first contraction served as the ‘practice’ contraction and was excluded from the MVIC calculation. In all cases, when a practice contraction was performed, only one was completed per participant. This single contraction was used to familiarize participants with the effort and movement required to perform an MVIC.

After performing the first set of MVICs, participants were given five minutes of rest to fully recover from the exertions. The HUMAC NORM was subsequently repositioned for the next set of contractions. This was repeated three times in total, such that participants had completed 15 MVICs (five elbow flexion, five knee extension, five hand grip). At all sessions, the same two researchers provided verbal encouragement throughout contractions to maintain consistency. For knee and elbow exertions, live visual feedback was provided *via* the HUMAC NORM. However, there was no visual feedback during the hand grip trials due to the outward facing gauge of the JAMAR hand grip dynamometer.

### Data analysis

**Figure 3:**
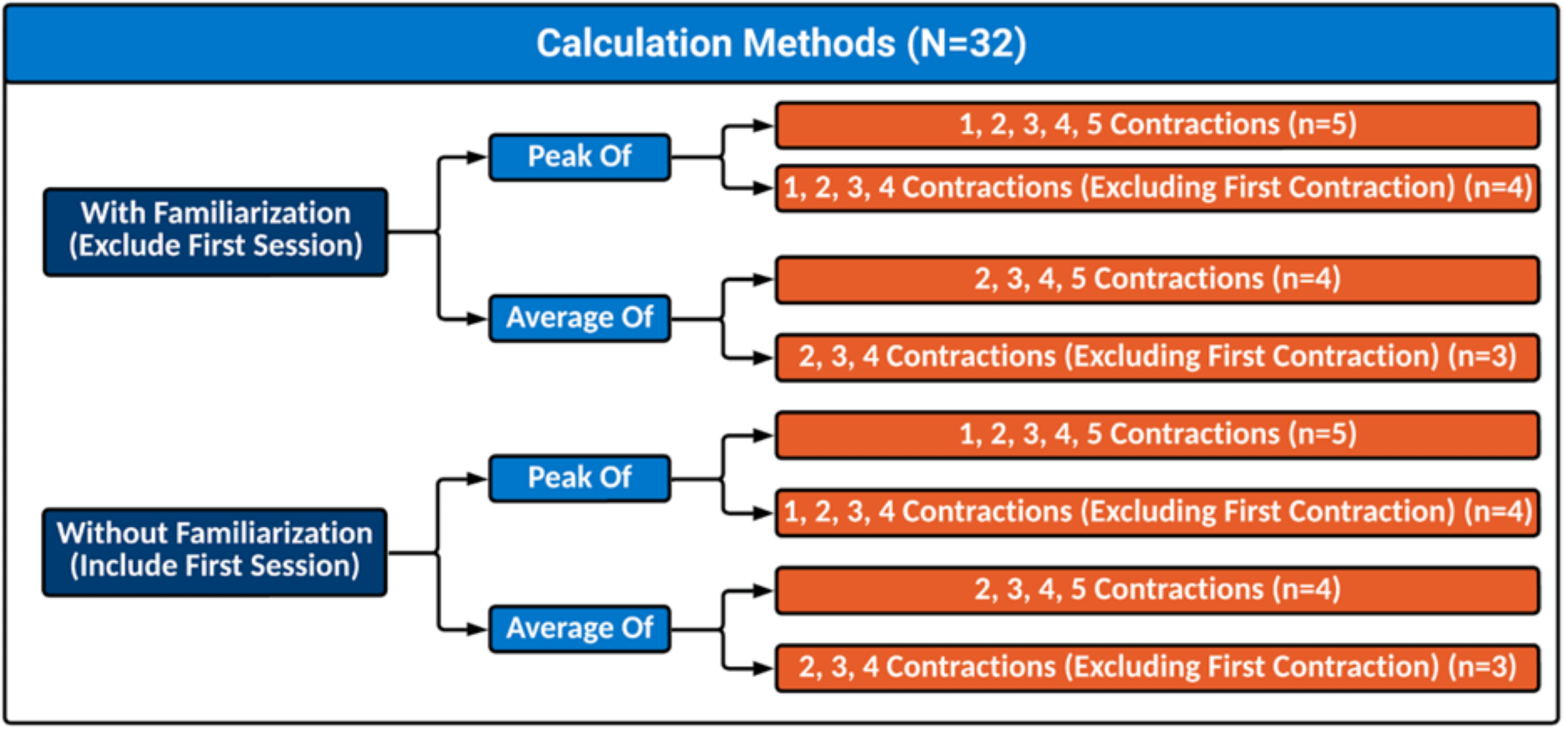
Visual depiction of all calculation methods evaluated. Outline of all calculation methods used to determine maximal strength.

Peak torque (N⋅m) values were obtained from the isokinetic dynamometer for elbow and knee exertions, while peak force (kg) was obtained from the hand dynamometer for hand grip trials. Muscle fatigue is commonly defined as a transient reduction in force-generating capacity (Enoka & Duchateau, 2008). In this study, muscle fatigue was evaluated by assessing whether strength decreased from contractions 1 through 5 within each session. Between-day strength values were analyzed to determine whether MVICs improved across sessions, indicating a learning effect. The present study explored 32 different methods of calculating baseline MVIC (Fig. 3). The first 18 methods of calculation included: (1) averaging two, three, four, or five MVICs, and (2) taking the peak of one, two, three, four, or five MVICs. The other 14 methods excluded the first MVIC, to represent providing a practice contraction, but were otherwise the same. All the aforementioned methods of calculating baseline maximal strength were conducted one with the inclusion of their first session as the without familiarization group and excluding the first session to demonstrate the use of a familiarization session. Familiarization sessions can be described as a session prior to data collections where participants perform the entire experimental protocol to be more comfortable with the data collection process.

### Statistical analysis

In this study, we employed a linear mixed-effects modelling approach to examine the effects of session and contraction number on MVIC strength for the three exertion types. Restricted Maximum Likelihood (REML) estimation was used to fit separate models for each contraction type (elbow flexion, hand grip, and knee extension). The fixed effects of interest included session number and contraction number, representing the within-subject factors hypothesised to influence MVIC strength. Participant ID was included as a random effect to account for repeated measures on the same participants, with random intercepts specified for each participant. Additionally, random slopes were permitted for session number to capture potential individual-specific variations in the relationship between session number and MVIC strength, which provided the best model fit according to Akaike information criterion (AIC). Model assumptions, such as normality of residuals and homoscedasticity, were assessed and met.

To determine relative between-day reliability for each contraction type and MVIC calculation method (*e.g.*, peak, average, familiarised, *etc.*), intraclass correlation coefficient (ICC) estimates and their 95% confidence intervals were calculated based on separate single-rating, absolute agreement, two-way mixed effects model ICC (3,1), as per recommendations by Koo & Li (2016). ICCs were interpreted using guidelines by Koo & Li (2016). Significance for this test was set at *p* < .05. ICC values of 0.5 or less indicate ‘Poor’ reliability, between 0.5 and 0.75 indicate ‘Moderate’ reliability, 0.75 and 0.9 indicate ‘Good’ reliability, and 0.9 or greater indicate ‘Excellent’ reliability. The same methodology was used to calculate relative within-day reliability between all contractions within each session.

To determine absolute between-day reliability, the standard error of measure (SEM) and minimum difference to be considered real (MD_95_) were both calculated. SEM indicates the day-to-day noise, whereas MD_95_ indicates the amount of change a measure must have such that it is a true change and not by chance, with 95% confidence. SEM was calculated by taking the root of the mean squared error (Weir, 2005). MD_95_ was calculated by taking the SEM multiplied by 1.96 multiplied by the square root of 2 (Weir, 2005). SEM was also calculated as a percentage of the grand mean by taking the SEM and dividing it by the grand mean. All statistical analyses were performed using R (version 4.0.3; R Core Team, 2020) and RStudio (v4.3.0; RStudio Team, 2023) statistical software.

## Results

### MVIC data

Table 2 shows all the MVIC values averaged across all participants for each contraction type (elbow flexion, knee extension, hand grip) for each session and contraction number.

**Table 2:** Average MVIC values and their standard deviations per contraction per session. Average maximum voluntary isometric contraction (MVIC) values ± standard deviations for elbow flexion, hand grip, and knee extension across four sessions.

Session number	Session 1					Session 2					Session 3					Session 4				
Exertion number	1	2	3	4	5	1	2	3	4	5	1	2	3	4	5	1	2	3	4	5
Elbow flexion (N⋅m)	57.8 ± 27.6	56.0 ± 36.8	55.5 ± 27.7	54.1 ± 27.2	53.8 ± 26.8	54.8 ± 24.0	54.0 ± 23.6	53.1 ± 22.1	52.3 ± 21.6	51.2 ± 20.3	54.0 ± 21.0	53.4 ± 22.0	54.1 ± 22.0	53.1 ± 21.4	54.8 ± 2.7	56.5 ± 23.4	54.6 ± 21.6	54.5 ± 21.5	54.4 ± 22.8	54.8 ± 23.0
Hand grip (kg)	38.2 ± 11.8	38.2 ± 11.5	38.0 ± 12.5	37.1 ± 13.5	36.4 ± 11.9	37.9 ± 12.0	37.1 ± 11.1	36.5 ± 12.6	37.6 ± 11.6	36.4 ± 11.9	38.2 ± 13.1	37.3 ± 12.0	36.0 ± 11.0	36.6 ± 10.4	36.2 ± 10.9	36.7 ± 12.3	35.7 ± 10.8	36.6 ± 13.0	36.1 ± 12.9	36.8 ± 12.9
Knee extension (N⋅m)	218 ± 75.2	213 ± 68.2	222 ± 67.6	226 ± 67.4	223 ± 67.9	233 ± 73.7	227 ± 75.7	228 ± 71.8	232 ± 81.8	225 ± 77.2	230 ± 84.7	230 ± 73.9	225 ± 73.4	226 ± 73.4	227 ± 67.9	220 ± 69.5	212 ± 54.9	214 ± 54.2	211 ± 54.1	214 ± 57.8

### Reliability measures

Relative within-day reliability was classified as ‘Excellent’ for the elbow flexion, knee extension, and hand grip MVIC types. Similarly, absolute reliability was strong with low Standard Error of Measurement (SEM) and Minimal Detectable Change at the 95% confidence level (MD_95_), indicating precise measurement and a good sensitivity to change within each session (Table 3).

**Table 3:** Relative and absolute within-session reliability metrics for elbow flexion, knee extension, and hand grip across four sessions. Reliability is reported as intraclass correlation coefficients (ICC), standard error of measurement (SEM), and minimal detectable difference at the 95% confidence level (MD_95_).

	Session 1	Session 2	Session 3	Session 4
**Elbow flexion**				
ICC	0.98	0.97	0.98	0.98
SEM (Nm)	4.29	4.15	3.19	3.03
MD_95_ (Nm)	11.8	11.5	8.86	8.40
**Knee extension**				
ICC	0.94	0.94	0.96	0.95
SEM (Nm)	16.7	18.9	14.6	12.9
MD_95_ (Nm)	46.5	52.4	40.5	35.7
**Hand grip**				
ICC	0.96	0.96	0.94	0.95
SEM (kg)	2.45	2.29	2.84	2.81
MD_95_ (kg)	6.80	6.36	7.89	7.80

**Table 4:** Between-day reliability (ICC, SEM%, MD95%) across calculation methods. Between-day intraclass correlation coefficients (ICC) were calculated for each calculation method using a single-rating, absolute-agreement, two-way mixed-effects model. The standard error of measurement (SEM) and the minimal detectable change at the 95% confidence level (MD95) are reported as percentages of the grand mean.

						ICC 95% CI				
Peak or average?	Familiarisation session?	Practice contraction?	MVIC test	Number of MVICs	ICC	Lower bound	Upper bound	*F*- Statistic	SEM%	MD95%
Peak	Yes	No	Elbow Flexion	1	0.95	0.89	0.98	57	9.38%	26.0%
Peak	Yes	No	Elbow Flexion	2	0.96	0.91	0.98	65	8.72%	24.2%
Peak	Yes	No	Elbow Flexion	3	0.96	0.92	0.98	73	8.16%	22.6%
Peak	Yes	No	Elbow Flexion	4	0.96	0.92	0.98	78	7.85%	21.8%
Peak	Yes	No	Elbow Flexion	5	0.97	0.93	0.99	89	7.45%	20.6%
Peak	Yes	No	Knee Extension	1	0.88	0.76	0.95	23	11.72%	32.5%
Peak	Yes	No	Knee Extension	2	0.89	0.78	0.95	25	10.82%	30.0%
Peak	Yes	No	Knee Extension	3	0.89	0.78	0.95	26	10.45%	29.0%
Peak	Yes	No	Knee Extension	4	0.9	0.79	0.96	29	10.29%	28.5%
Peak	Yes	No	Knee Extension	5	0.89	0.79	0.96	29	10.43%	28.9%
Peak	Yes	No	Hand Grip	1	0.93	0.85	0.97	40	8.86%	24.6%
Peak	Yes	No	Hand Grip	2	0.93	0.86	0.97	43	8.21%	22.8%
Peak	Yes	No	Hand Grip	3	0.95	0.9	0.98	62	6.93%	19.2%
Peak	Yes	No	Hand Grip	4	0.96	0.91	0.98	69	6.52%	18.1%
Peak	Yes	No	Hand Grip	5	0.96	0.91	0.98	65	6.67%	18.5%
Peak	No	No	Elbow Flexion	1	0.93	0.86	0.97	54	11.57%	32.1%
Peak	No	No	Elbow Flexion	2	0.93	0.87	0.97	59	10.98%	30.4%
Peak	No	No	Elbow Flexion	3	0.93	0.87	0.97	59	11.00%	30.5%
Peak	No	No	Elbow Flexion	4	0.94	0.88	0.97	61	10.79%	29.9%
Peak	No	No	Elbow Flexion	5	0.95	0.9	0.98	79	9.54%	26.4%
Peak	No	No	Knee Extension	1	0.88	0.78	0.95	32	11.52%	31.9%
Peak	No	No	Knee Extension	2	0.9	0.81	0.96	37	10.46%	29.0%
Peak	No	No	Knee Extension	3	0.9	0.81	0.96	36	10.18%	28.2%
Peak	No	No	Knee Extension	4	0.92	0.84	0.96	47	9.34%	25.9%
Peak	No	No	Knee Extension	5	0.91	0.84	0.96	46	9.40%	26.1%
Peak	No	No	Hand Grip	1	0.93	0.86	0.97	51	8.86%	24.6%
Peak	No	No	Hand Grip	2	0.93	0.87	0.97	56	8.26%	22.9%
Peak	No	No	Hand Grip	3	0.95	0.89	0.98	71	7.37%	20.4%
Peak	No	No	Hand Grip	4	0.96	0.92	0.98	91	6.53%	18.1%
Peak	No	No	Hand Grip	5	0.96	0.92	0.98	94	6.40%	17.7%
Average	Yes	No	Elbow Flexion	2	0.97	0.93	0.99	94	7.31%	20.2%
Average	Yes	No	Elbow Flexion	3	0.97	0.94	0.99	106	6.81%	18.9%
Average	Yes	No	Elbow Flexion	4	0.98	0.95	0.99	121	6.39%	17.7%
Average	Yes	No	Elbow Flexion	5	0.98	0.96	0.99	154	5.77%	16.0%
Average	Yes	No	Knee Extension	2	0.88	0.76	0.95	24	11.08%	30.7%
Average	Yes	No	Knee Extension	3	0.88	0.77	0.95	25	10.68%	29.6%
Average	Yes	No	Knee Extension	4	0.88	0.77	0.95	26	10.64%	29.5%
Average	Yes	No	Knee Extension	5	0.89	0.77	0.95	26	10.40%	28.8%
Average	Yes	No	Hand Grip	2	0.94	0.87	0.97	48	7.86%	21.8%
Average	Yes	No	Hand Grip	3	0.95	0.9	0.98	63	6.86%	19.0%
Average	Yes	No	Hand Grip	4	0.96	0.91	0.98	68	6.58%	18.2%
Average	Yes	No	Hand Grip	5	0.96	0.91	0.98	69	6.51%	18.1%
Average	No	No	Elbow Flexion	2	0.95	0.89	0.98	70	10.09%	28.0%
Average	No	No	Elbow Flexion	3	0.95	0.9	0.98	72	9.90%	27.4%
Average	No	No	Elbow Flexion	4	0.95	0.9	0.98	73	9.85%	27.3%
Average	No	No	Elbow Flexion	5	0.95	0.91	0.98	80	9.44%	26.2%
Average	No	No	Knee Extension	2	0.89	0.79	0.95	34	10.81%	30.0%
Average	No	No	Knee Extension	3	0.89	0.79	0.95	35	10.39%	28.8%
Average	No	No	Knee Extension	4	0.9	0.81	0.96	38	9.95%	27.6%
Average	No	No	Knee Extension	5	0.9	0.81	0.96	39	9.67%	26.8%
Average	No	No	Hand Grip	2	0.94	0.88	0.97	61	7.96%	22.1%
Average	No	No	Hand Grip	3	0.95	0.9	0.98	79	7.08%	19.6%
Average	No	No	Hand Grip	4	0.96	0.91	0.98	91	6.63%	18.4%
Average	No	No	Hand Grip	5	0.96	0.92	0.98	97	6.37%	17.7%
Peak	Yes	Yes	Elbow Flexion	1	0.97	0.94	0.99	99	7.15%	19.8%
Peak	Yes	Yes	Elbow Flexion	2	0.97	0.94	0.99	97	7.08%	19.6%
Peak	Yes	Yes	Elbow Flexion	3	0.97	0.94	0.99	106	6.74%	18.7%
Peak	Yes	Yes	Elbow Flexion	4	0.97	0.93	0.99	87	7.50%	20.8%
Peak	Yes	Yes	Knee Extension	1	0.85	0.7	0.93	18	12.15%	33.7%
Peak	Yes	Yes	Knee Extension	2	0.87	0.74	0.94	22	10.87%	30.1%
Peak	Yes	Yes	Knee Extension	3	0.86	0.73	0.94	22	11.52%	31.9%
Peak	Yes	Yes	Knee Extension	4	0.85	0.71	0.94	20	11.94%	33.1%
Peak	Yes	Yes	Hand Grip	1	0.91	0.82	0.96	32	9.22%	25.6%
Peak	Yes	Yes	Hand Grip	2	0.96	0.92	0.98	76	6.27%	17.4%
Peak	Yes	Yes	Hand Grip	3	0.96	0.92	0.98	75	6.18%	17.1%
Peak	Yes	Yes	Hand Grip	4	0.96	0.91	0.98	68	6.46%	17.9%
Peak	No	Yes	Elbow Flexion	1	0.94	0.89	0.97	65	10.48%	29.0%
Peak	No	Yes	Elbow Flexion	2	0.94	0.88	0.97	60	10.77%	29.9%
Peak	No	Yes	Elbow Flexion	3	0.94	0.88	0.97	64	10.41%	28.8%
Peak	No	Yes	Elbow Flexion	4	0.95	0.9	0.98	76	9.61%	26.6%
Peak	No	Yes	Knee Extension	1	0.85	0.73	0.93	25	12.12%	33.6%
Peak	No	Yes	Knee Extension	2	0.88	0.78	0.95	32	10.46%	29.0%
Peak	No	Yes	Knee Extension	3	0.89	0.79	0.95	36	10.25%	28.4%
Peak	No	Yes	Knee Extension	4	0.89	0.79	0.95	34	10.45%	29.0%
Peak	No	Yes	Hand Grip	1	0.91	0.83	0.96	43	9.08%	25.2%
Peak	No	Yes	Hand Grip	2	0.95	0.91	0.98	84	6.93%	19.2%
Peak	No	Yes	Hand Grip	3	0.96	0.93	0.98	105	6.11%	16.9%
Peak	No	Yes	Hand Grip	4	0.96	0.92	0.98	98	6.21%	17.2%
Average	Yes	Yes	Elbow Flexion	2	0.97	0.95	0.99	116	6.52%	18.1%
Average	Yes	Yes	Elbow Flexion	3	0.98	0.95	0.99	136	6.03%	16.7%
Average	Yes	Yes	Elbow Flexion	4	0.98	0.96	0.99	167	5.59%	15.5%
Average	Yes	Yes	Knee Extension	2	0.86	0.73	0.94	21	11.29%	31.3%
Average	Yes	Yes	Knee Extension	3	0.86	0.73	0.94	22	11.37%	31.5%
Average	Yes	Yes	Knee Extension	4	0.87	0.74	0.94	22	11.09%	30.7%
Average	Yes	Yes	Hand Grip	2	0.94	0.88	0.98	49	7.71%	21.4%
Average	Yes	Yes	Hand Grip	3	0.95	0.9	0.98	58	7.02%	19.5%
Average	Yes	Yes	Hand Grip	4	0.95	0.9	0.98	59	7.01%	19.4%
Average	No	Yes	Elbow Flexion	2	0.94	0.89	0.98	67	10.27%	28.5%
Average	No	Yes	Elbow Flexion	3	0.94	0.89	0.98	69	10.16%	28.2%
Average	No	Yes	Elbow Flexion	4	0.95	0.9	0.98	75	9.75%	27.0%
Average	No	Yes	Knee Extension	2	0.87	0.76	0.94	32	10.98%	30.4%
Average	No	Yes	Knee Extension	3	0.88	0.78	0.95	33	10.47%	29.0%
Average	No	Yes	Knee Extension	4	0.89	0.79	0.95	34	10.12%	28.0%
Average	No	Yes	Hand Grip	2	0.94	0.88	0.97	64	7.82%	21.7%
Average	No	Yes	Hand Grip	3	0.95	0.91	0.98	81	6.98%	19.4%
Average	No	Yes	Hand Grip	4	0.96	0.91	0.98	86	6.75%	18.7%

**Figure 4:**
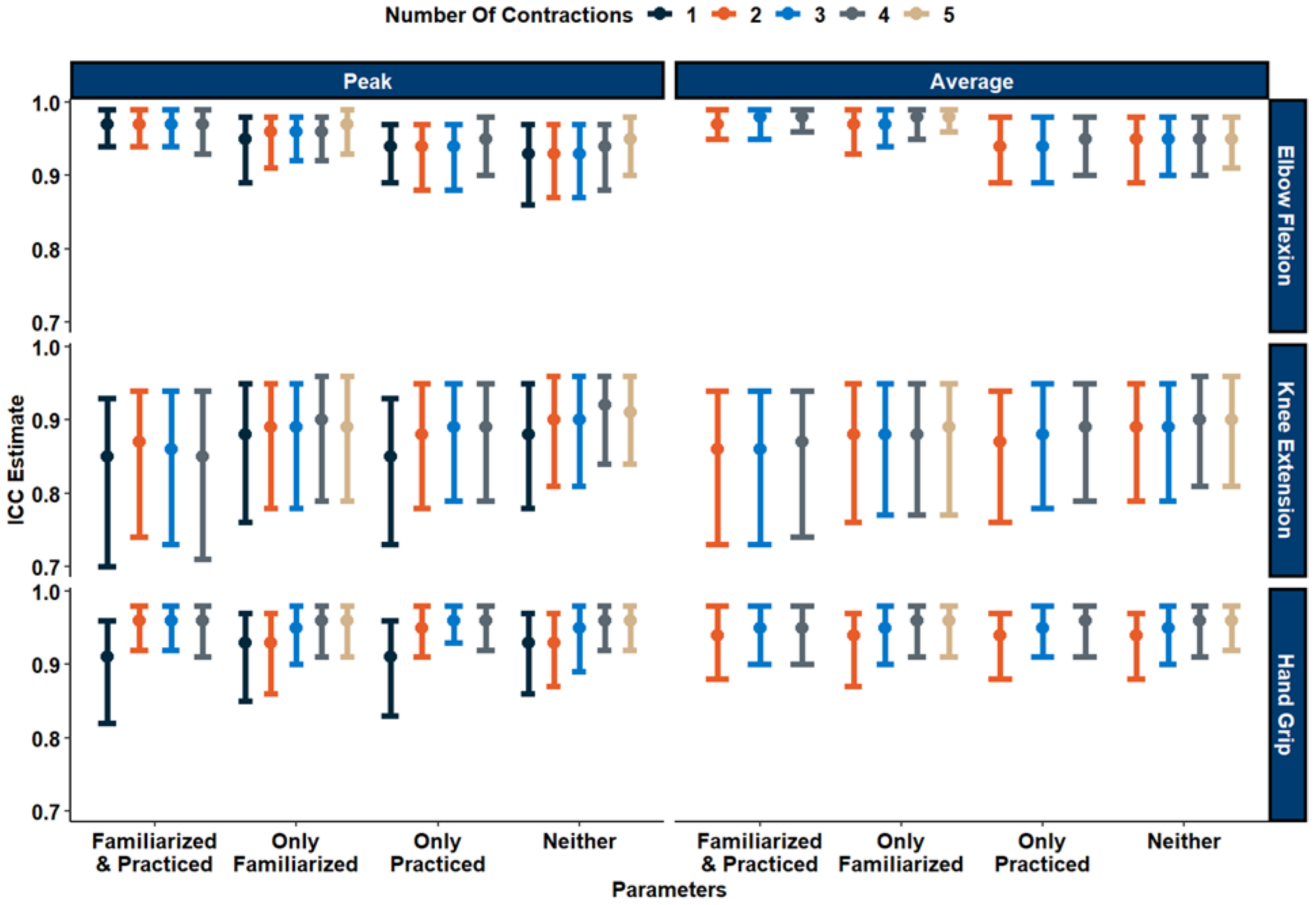
ICC estimates and their 95% confidence interval for elbow flexion, knee extension, and hand grip. Each data point represents the Intraclass Correlation Coefficient (ICC) estimate for reliability of strength measurements across different conditions and number of contractions. Different colours indicate the number of contractions used (Dark blue - 1, Orange - 2, Light Blue - 3, Gray - 4, Beige- (5), and vertical bars represent 95% confidence intervals.

**Figure 5:**
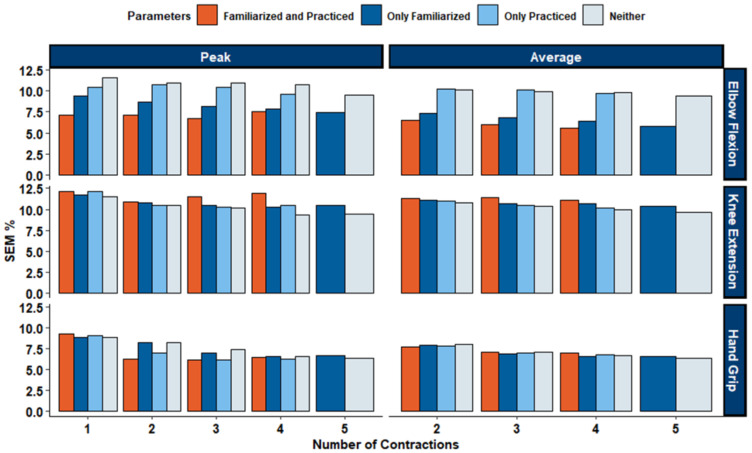
SEM% of grand mean for elbow flexion, knee extension, and hand grip. Bars represent different levels of preparation: familiarized and practiced (orange), only familiarized (dark blue), only practiced (light blue), and neither (gray). Lower SEM% indicates greater measurement reliability. Results show that both familiarization and practice generally reduce variability, particularly for elbow flexion peak values, while hand grip demonstrates the lowest SEM% across conditions.

**Figure 6:**
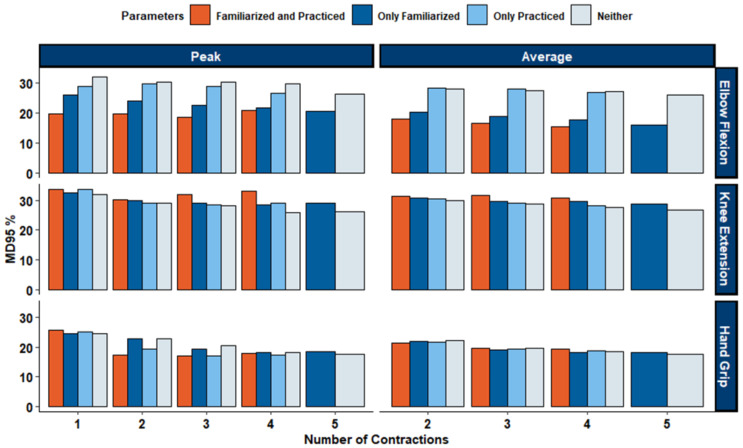
MD_95%_ of grand mean for elbow flexion, knee extension and hand grip. Bars represent different levels of preparation: familiarized and practiced (orange), only familiarized (dark blue), only practiced (light blue), and neither (gray). Lower MD95% indicates greater measurement sensitivity. Elbow flexion shows higher variability compared with knee extension and hand grip, while combined familiarization and practice consistently reduce MD95% across contraction trials.

All ICC ratings (Fig. 4) for elbow flexion MVICs produced ‘Excellent’ between-day reliability, with an average ICC rating of 0.96 across all calculation methods (ICC: [0.93−0.98]). Absolute between-day reliability was high with an average SEM of 8.72% (range of 5.59%–11.6%), and an average MD_95_ of 24.2% (range of 15.5%–32.1%). Between-day reliability for the hand grip assessment produced ‘Excellent’ between-day reliability, with an average ICC rating of 0.95 across all calculation methods (ICC: [0.91−0.96]). Absolute between-day reliability was high with an average SEM of 8.72% (range of 5.59%–11.6%), and an average MD_95_ of 24.2% (range of 15.5%–32.1%). For the knee extension MVIC test, between-day reliability varied from ‘Good’ to ‘Excellent’, with an average ICC rating of 0.88 across all calculation methods (ICC: [0.85−0.92]). Absolute between-day reliability was high with an average SEM of 10.73% (range of 9.34%–12.2%), and an average MD_95_ of 29.7% (range of 25.9%–33.7%). ICC, SEM%, and MD_95_% values for all MVIC calculation methods can be found in Table 4. Visualizations for all SEM can be found in Fig. 5, and visualizations for MD_95_ can be found in Fig. 6.

### Fatigue effects on MVIC strength

In our study, the strongest contraction strength was found to occur most frequently during the first exertion, accounting for approximately 38.4% of instances. Subsequently, during the second exertion, this peak exertion was observed in approximately 14.8% of cases. The occurrence of peak exertion increased slightly during the third exertion, representing around 15.3% of observations. Conversely, peak exertion during the fourth and fifth exertions accounted for approximately 16.2% and 15.3% of occurrences, respectively.

**Figure 7:**
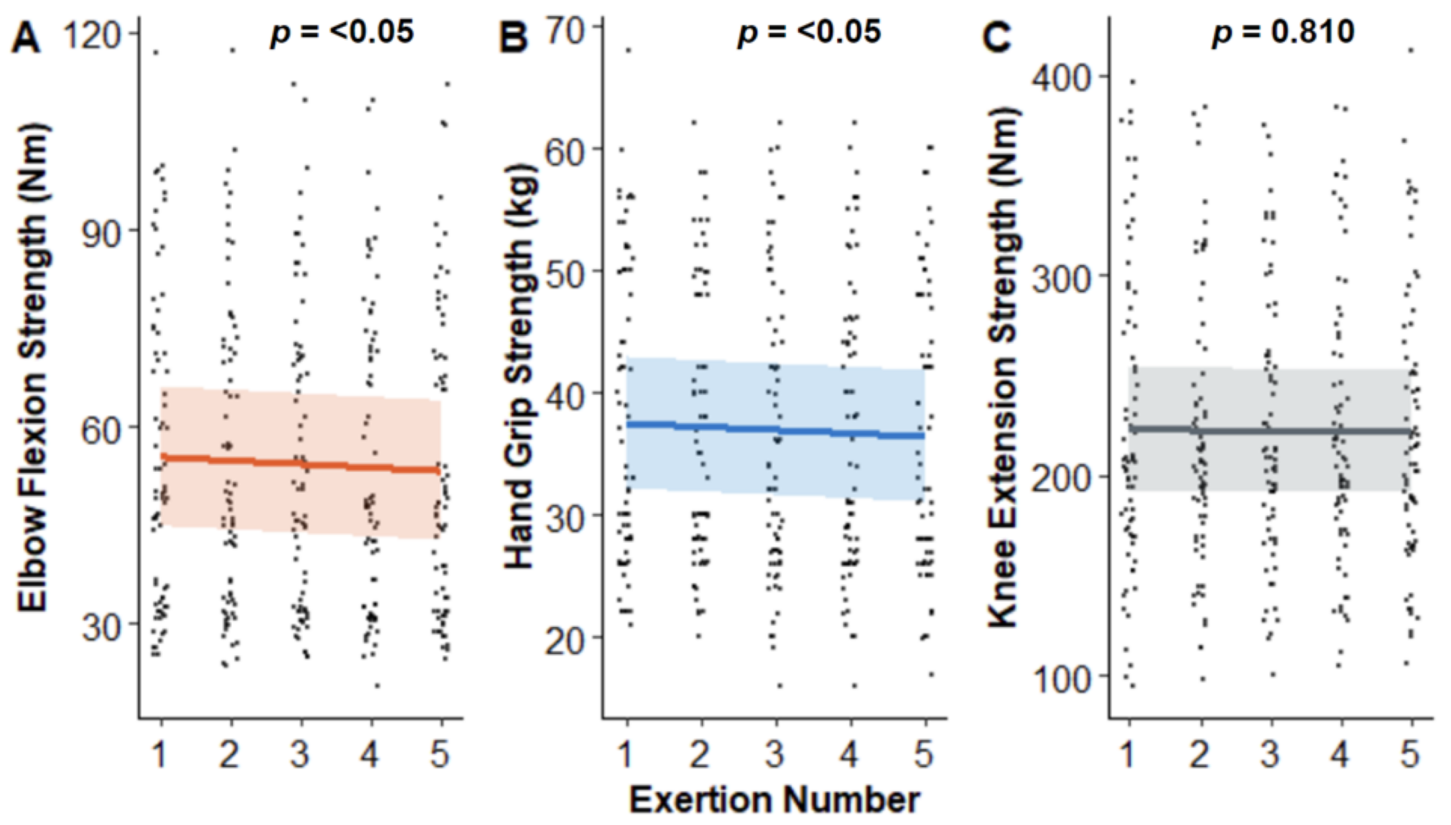
Contraction strength per exertion for each contraction type. Each dot on the graph represents a single contraction. Coloured lines depict the linear regression, with the shading indicating the 95% confidence interval.

The linear mixed-effects model examining MVIC strength for elbow flexion revealed exertion number (estimate = −0.53, SE = 0.16, *p* = 0.00076) significantly impacted MVIC strength (Fig. 7A), indicating that MVIC strength progressively declines as a participant performs more exertions. More precisely, there was a reduction of 0.5 Nm (1.1% of mean) for elbow flexion and 0.27 kg (0.73% of mean) for hand grip exertions with each contraction executed. The session number did not significantly affect elbow flexion strength (estimate = −0.06, SE = 0.81, *p* = 0.941). Similarly, hand grip analysis also revealed significant associations (Fig. 7B). Exertion number significantly influenced grip strength (estimate = −0.2708, SE = 0.106, *p* = 0.0106), indicating a decrease in grip strength with increasing exertion number. However, the session number again did not have a significant effect (estimate = −0.386, SE = 0.310, *p* = 0.2129). Unexpectedly, the linear mixed-effects model examining knee extension MVIC revealed that neither session number (estimate = −1.9741, SE = 2.4654, t = −0.801, *p* = 0.423) nor exertion number (estimate = −0.1931, SE = 0.8033, t = −0.240, *p* = 0.810) had a significant effect on knee extension strength (Fig. 7C).

## Discussion

The present study aimed to investigate the within- and between-day reliability of measurements for elbow flexion, knee extension, and hand grip Maximum Voluntary Isometric Contractions (MVICs). This study also examined whether force generation capacity was modified by the number of MVICs performed. Our findings demonstrate excellent within-day reliability for all three MVIC types, as evidenced by high Intraclass Correlation Coefficients (ICCs) ranging from 0.94 to 0.98. These results suggest strong agreement between repeated measurements within the same day, indicating the consistency and stability of the three MVIC types (*i.e.,* elbow flexion, knee extension, and hand grip). These findings align with previous research indicating that MVIC measurements for elbow flexion (Howatson & Van Someren, 2005), knee extension (Al-Zahrani et al., 2009), and hand grip (Lagerström & Nordgren, 1996) are reliable and suitable for assessing muscle strength within a single testing session.

Moreover, the study extends its scope to assess between-day reliability of baseline MVIC measurements, which are commonly used across various scientific disciplines. Between-day reliability was assessed for a variety of baseline MVIC calculation methods, specifically: averaging two, three, four, or five MVICs, or using the peak of one, two, three, four, or five MVICs; both with and without a separate familiarisation session containing only practice contractions and/or single practice contraction day of data collection factored in. ‘Excellent’ to ‘Good’ reliability was seen across all MVIC tests and all methods of calculating baseline MVIC in the study. These reliability scores, being somewhat consistent between each MVIC test posture, may be indicative of the innately ‘Excellent’ relative within-session reliability of the MVIC types measured in this study. Elbow flexion MVICs had substantial increases in relative and absolute between-day reliability due to a familiarisation session or practice contraction, while smaller increases occurred for the hand grip MVICs. Unexpectedly, the reliability of knee extension exhibited a slight decline when incorporating familiarisation sessions and practice contractions into the calculation methodology. Prior research has indicated that the between-session reliability of MVIC strength can be improved by providing a familiarisation session during knee extension (Al-Zahrani et al., 2009), and neck flexion strength tests (Strimpakos et al., 2004). In the present study, similar reliability between the peak and average calculation methods was found. Consequently, the peak should likely be used as it is more representative of maximal exertion (Roberts et al., 2011). Although there was a tendency for between-session reliability to improve with an increase in the number of contractions across all types of MVIC, it is noteworthy that ‘Good’ to ‘Excellent’ reliability was consistently observed across all calculation methods, even using a single contraction.

In addition to evaluating relative reliability, we assessed the absolute between-day reliability of baseline MVIC measurements to quantify the degree of day-to-day variability inherent in common MVIC protocols. The Standard Error of Measurement (SEM), which represents the typical day-to-day variability in each MVIC test, ranged from approximately 8–11%, indicating modest absolute measurement error. The MD95, which is directly derived from the SEM, therefore reflects the minimum amount of change required to exceed this measurement error with 95% confidence for an individual. Although the MD95 values ranged from ∼24–30%, it is important to emphasize that these values reflect the statistical precision of the protocol rather than the magnitude of change required for a musculoskeletal intervention to be considered clinically meaningful. When considered alongside typical strength gains reported in musculoskeletal intervention programs, ∼20–40% in untrained or moderately trained individuals and ∼5–10% in trained athletes (Iwasaki et al., 2006), the present protocol remains sufficiently sensitive to detect clinically meaningful improvements. Notably, the MD95 values reported here align with those observed in other dynamometry protocols. For example, dynamic knee extensor testing has demonstrated SEM% values of ∼15–23%, corresponding to an MD95 of ∼65% for a 167 Nm contraction (Habets et al., 2018), while isometric shoulder rotational assessments have shown MD95 ranges of ∼17–30% (Holt et al., 2016; Johansson et al., 2015). Meaningful physiological or clinical improvements smaller than the MD95 may still occur; however, such changes would not exceed the threshold for detectable change with 95% confidence for an individual using this specific protocol. This distinction underscores that MD95 reflects measurement precision and statistical detectability, whereas clinical meaningfulness depends on the expected magnitude of adaptation from an intervention.

While increasing the number of contractions yields only modest gains in reliability, the mixed-effects models indicate a notable trade-off. Namely, there is a gradual decrease in MVIC strength with each additional exertion for both elbow flexion and hand grip assessments. This decrease in MVIC strength implies the accumulation of fatigue during consecutive contractions, resulting in diminished muscle strength. More specifically, with each contraction performed, strength decreased significantly by 0.5 Nm (1.10% of mean) and 0.27 kg (0.73% of mean) for the elbow flexion and hand grip exertions, respectively. These fatigue effects suggest that fewer contractions may be advantageous when aiming to capture an individual’s true maximum strength. Fatigue-related experimental protocols may be particularly sensitive, as pre-fatiguing participants with excessive baseline contractions could potentially confound results. This discrepancy is likely related to task design and muscle-specific physiology. The quadriceps have greater muscle mass and force-generating capacity than the elbow flexors or hand muscles, and may require longer contraction durations or higher overall volume before fatigue becomes apparent (Enoka & Duchateau, 2016). Under the relatively brief, intermittent MVIC protocol used here (five 5-s contractions with 1-min rest), it is therefore plausible that knee extensor fatigue did not reach a level that produced a measurable decline in torque. The present paper results can be further supported by prior reliability studies showing that, although knee extensor MVICs typically demonstrate excellent between-day reliability (ICC > 0.90; Al-Zahrani et al., 2009), their reliability is often slightly lower than that of elbow flexion (ICC > 0.95; Howatson & Van Someren, 2005) and hand grip assessments (ICC > 0.95; Lagerström & Nordgren, 1998). Such muscle-specific differences in reliability and fatigue resistance are consistent with the present findings, wherein the quadriceps exhibited minimal within-session fatigue despite repeated maximal efforts. Notably, session number did not significantly affect MVIC strength in any of the MVIC tests, indicating that fatigue-related effects are primarily influenced by the number of exertions. This can be explained by the shorter period it takes to recover from intense short contractions, such as the MVICs performed in this study, compared to the 48 h of rest provided to participants (Carroll, Taylor & Gandevia, 2017).

The between-day reliability and fatigue results of the present study hint that the trend of performing multiple MVICs to determine a baseline MVIC value is not ideal. With only one contraction, and no familiarisation session or practice contraction prior, relative between-day reliability was ‘Good’ to ‘Excellent’ across all MVIC types (elbow flexion, hand grip, and knee extension). Considering the significant effect of exertion number on strength, and high ICCs between sessions, taking one MVIC is likely sufficient to model subsequent submaximal contractions. Previous studies have found similar results for hand grip, specifically indicating one MVIC to be enough (Lagerström & Nordgren, 1998; Mathiowetz, 1990). If there is a notable concern about fatigue before an experimental protocol, our findings suggest that a single MVIC value can be used throughout the study’s duration, provided no additional MVICs are required for other experimental measures, such as electromyography. This approach avoids requiring participants to perform maximal contractions at the beginning of each data collection session for a study. However, this recommendation should only be considered in studies that keep a similar level of control (*e.g.*, sessions at a consistent time of day, controlling outside exercise exposure, detailed recording of dynamometer positions and participant postures, *etc.*).

The results of the present study should be interpreted in light of several limitations. For instance, results from this study may not be generalizable to older populations, as the experiment was performed on university students. Additionally, the sample was relatively homogeneous in age, health status, and physical activity characteristics, which may have reduced between-subject variability and contributed to the high reliability estimates observed. Although a 1-minute rest interval between contractions was used, drawing on previous literature (Abdel-Malek et al., 2022; Kan, Dundas & Nosaka, 2013; Todd, Gorman & Gandevia, 2004), longer rest periods (*e.g.*, 2 min) may have mitigated the small fatigue effects observed. Future studies could systematically examine the influence of different rest intervals on force recovery and fatigue development during repeated MVIC testing. This study quantified MVICs without the use of electromyography (EMG). As a result, the procedures evaluated here should not be used as recommended protocols for EMG normalization. For studies requiring EMG-based normalization or assessment of maximal neural drive, researchers should refer to the consensus for experimental design in electromyography (CEDE) consensus recommendations (Besomi et al., 2020). Furthermore, only three different postures/muscles were tested in this study, all of which produced marginally different between- and within-day reliability. Consequently, the reliability of the MVIC postures used in this study may not be directly translatable to untested postures. However, considering the homogeneity of the reliability measures across all test postures in this study, marked by ‘Good’ to ‘Excellent’ relative reliability, it is probable that MVIC tests for other joints coexist within this reliability range. Hence, methodological decisions can be derived from these data in the absence of more specific reliability information.

## Conclusions

As hypothesized, different calculation methods produced varying levels of between-session reliability, ranging from ‘Good’ to ‘Excellent’ relative between-day reliability, regardless of the number of contractions. Given this, and the propensity for MVIC strength to decline with increasing contraction numbers, it is recommended to take the peak of one contraction. Contrary to our hypothesis, the presence of a practice contraction and/or familiarisation had mixed effects on reliability, depending on the specific MVIC test, generally increasing reliability for the elbow flexion MVICs only. Lastly, as hypothesized, maximal strength progressively decreases for elbow and hand grip MVICs as additional contractions were performed. Researchers have the flexibility to adjust the number of contractions based on a desired level of reliability, but they should be wary of pre-protocol fatigue development. Future studies may further investigate the optimal balance between reliability and participant fatigue, enhancing the utility of MVIC assessments in research and clinical settings. Additionally, although the present study was not designed to evaluate sex-specific reliability, future work could investigate whether sex influences the reliability of MVIC measurements.

##  Supplemental Information

10.7717/peerj.20848/supp-1 Supplemental Information 1 ANOVA and Mixed-Effects Models for Hand Grip Exertions R script performing data preprocessing, visualization, and statistical analysis of hand grip exertion trials. Includes boxplots, normality checks, repeated-measures ANOVA, mixed-effects modeling with lmerTest and buildmer, post-hoc comparisons with emmeans, and effect size calculations.

10.7717/peerj.20848/supp-2 Supplemental Information 2 ANOVA and Mixed-Effects Models for Knee Extension Exertions R script performing data preprocessing, visualization, and statistical analysis of Knee extension exertion trials. Includes boxplots, normality checks, repeated-measures ANOVA, mixed-effects modeling with lmerTest and buildmer, post-hoc comparisons with emmeans, and effect size calculations.

10.7717/peerj.20848/supp-3 Supplemental Information 3 Intraclass Correlation and Reliability Analysis of Knee, Elbow, and Hand Grip Strength Measurements with and without Familiarization R script calculating intraclass correlation coefficients (ICCs) and standard error of measurement (SEM) for knee, elbow, and grip maximum voluntary contraction datasets. Analyses are performed both with and without the first trial (familiarization) included.

10.7717/peerj.20848/supp-4 Supplemental Information 4 ANOVA and Mixed-Effects Models for Elbow Exertions R script performing data preprocessing, visualization, and statistical analysis of elbow flexion exertion trials. Includes boxplots, normality checks, repeated-measures ANOVA, mixed-effects modeling with lmerTest and buildmer, post-hoc comparisons with emmeans, and effect size calculations.

10.7717/peerj.20848/supp-5 Supplemental Information 5 Distribution of Peak Maximum Voluntary Contraction Across Exertion Numbers and Types R script that identifies the trial at which peak corrected MVC occurs for each participant, session, and exertion type. Summarizes frequency and proportion of peak exertions across all trials and by exertion type, and visualizes results using bar plots.

10.7717/peerj.20848/supp-6 Supplemental Information 6 Mixed-Effects Modeling of Exertion Number and Session Effects on Corrected MVC Across Exertion Types R script fitting linear mixed-effects models to examine how exertion number and session influence corrected MVC during elbow flexion, grip strength, and knee extension. Uses the buildmer package for model selection, diagnostic and effect plots for visualization, and arranges final effect plots into a combined figure.

10.7717/peerj.20848/supp-7 Supplemental Information 7 Results Table For Reliability of all Calculation Methods Each row reports reliability outcomes for maximum voluntary contraction (MVC) testing, including ICC values with 95% confidence intervals, SEM and SEM% as error estimates, and MD95 as the minimal detectable difference. Results are grouped by muscle, contraction type, and testing condition (with or without familiarization and practice contractions).

10.7717/peerj.20848/supp-8 Supplemental Information 8 Reliability Statistics for Baseline MVC Protocols The intraclass correlation coefficients (ICC) with 95% confidence intervals, standard error of measurement (SEM), and minimal detectable change (MD95) across different baseline MVC calculation methods. Methods vary by use of familiarization, peak or average contraction, practice trial inclusion, and number of MVC repetitions. Lower SEM and MD95 values indicate greater measurement consistency and precision

10.7717/peerj.20848/supp-9 Supplemental Information 9 Raw Participant MVC Trial Data The individual participant trial data for maximum voluntary contractions (MVC). Each row indicates participant ID, handedness, footedness, testing session, exertion type, and trial number. MVC values represent raw measurements, while Corrected MVC accounts for adjustments applied during data processing. These raw data formed the basis for reliability and group-level analyses.

## Additional Information and Declarations

### Data Availability

The following information was supplied regarding data availability: The code and data are available in the Supplemental Files.
